# What Is the Role of Diabetic Alert Dogs in Glycemic Monitoring for Individuals with Type 1 Diabetes? A Scoping Review

**DOI:** 10.3390/medsci14010039

**Published:** 2026-01-13

**Authors:** Nathalia Marçallo Peixoto Souza, Paula Rothbarth Silva, Christiane Mayrhofer Grocoske de Lima, Mateus Santana Lopes, Patricia Sthefani Calixto, Bruna Mariza Zampier Bilek, Luana Mota Ferreira, Marciane Welter, Fabiane Gomes de Moraes Rego, Marcel Henrique Marcondes Sari

**Affiliations:** PostGraduate Program in Pharmaceutical Sciences, Federal University of Paraná, Curitiba 80210-170, Paraná, Brazilrego@ufpr.br (F.G.d.M.R.)

**Keywords:** quality of life, assistance animals, hyperglycemia, hypoglycemia, non-invasive glucose monitoring

## Abstract

**Background/Objectives**: Type 1 diabetes mellitus (T1DM) is a chronic autoimmune condition that requires continuous glycemic monitoring to prevent acute and long-term complications. In recent years, Diabetic Alert Dogs (DADs) have been increasingly used as an adjunctive strategy to assist individuals with T1DM by alerting glycemic fluctuations through olfactory detection of physiological changes. Despite growing interest, the available evidence remains heterogeneous and fragmented. **Methods**: Therefore, this scoping review was conducted to address the following research question: “*What evidence is available regarding the relationship between Diabetic Alert Dogs (DADs) and glycemic monitoring in individuals with T1DM?*”, conducted in accordance with the Joanna Briggs Institute methodology and reported following the PRISMA Extension for Scoping Reviews. **Results**: Searches were performed in PubMed, Scopus, and Web of Science without time restrictions. After duplicate removal (*n* = 485), 2379 records were screened, of which 24 articles underwent full-text assessment and 10 studies met the predefined inclusion criteria. Regarding glycemic alteration detection, most studies (7/10) reported that DADs could identify both hypoglycemic and hyperglycemic episodes, while the remaining studies focused exclusively on hypoglycemia detection. Sensitivity values were consistently higher for hypoglycemia than for hyperglycemia, and none reported false alert rates exceeding 20%. In addition to glycemic alert performance, improvements in perceived safety, independence, and quality of life were described in half of the included studies (5/10). **Conclusions**: By systematically mapping the characteristics, outcomes, and methodological approaches of studies involving DADs, this scoping review provides an overview of current evidence and identifies key knowledge gaps in training protocols, outcome standardization, and performance reporting.

## 1. Introduction

According to the International Diabetes Federation (IDF), approximately 589 million people live worldwide with a diagnosis of diabetes mellitus (DM), a chronic condition characterized by persistently elevated circulating glucose levels due to insufficient insulin production or resistance to the hormone. Of these individuals, approximately 9.2 million are diagnosed with type 1 diabetes mellitus (T1DM), representing between 5 and 10% of diagnosed patients, and it is estimated that this number will exceed 14 million by the year 2050 [1]. Also known as insulin-dependent diabetes, T1DM is an autoimmune condition in which autoantibodies destroy pancreatic β-cells, leading to absolute insulin deficiency. This immune-mediated process may occur rapidly, as is more common in children, or progress more slowly, as observed in adults [2].

Glycemic control is essential for individuals living with T1DM, both for adjusting insulin dosages and for monitoring the frequency and severity of hypoglycemic and hyperglycemic episodes [3]. Currently, the two most widely used methods for assessing circulating glucose levels are capillary blood sampling via glucometer and continuous glucose monitoring (CGM) systems, which provide real-time glucose readings through a subcutaneous sensor [4]. Although these methods constitute the cornerstone of diabetes management, they are not without limitations. Capillary blood glucose testing is inherently invasive, requires repeated finger pricks, and depends on user adherence and technical proficiency, which may be particularly challenging for children, older adults, and individuals with reduced manual dexterity or cognitive limitations [5]. CGM systems, while less dependent on frequent finger-stick measurements, involve subcutaneous sensor insertion, incur acquisition and maintenance costs, and may be subject to technical failures, calibration errors, or signal delays [6,7]. Moreover, both approaches may fail to adequately capture asymptomatic, nocturnal, or rapidly fluctuating glycemic events, potentially contributing to patient anxiety, fear of hypoglycemia, and reduced confidence in glycemic self-management [8,9].

In light of the practical, technical, and emotional limitations of conventional glycemic monitoring methods, complementary support strategies that do not rely solely on user-operated technologies have become increasingly relevant. Within this context, service animals are widely recognized for their valuable roles in assisting individuals with health-related limitations that require continuous monitoring, early warning, and timely intervention. Examples include guide dogs supporting individuals with significant visual impairments, alert dogs assisting people with hearing loss, emotional support dogs aiding individuals with mental health conditions or post-traumatic stress disorders, and alert dogs helping patients with neurological conditions who may be at risk of fainting or seizures [10,11]. By providing real-time alerts based on behavioral or physiological cues, these animals can compensate for technological limitations, reduce dependence on constant user vigilance, and offer an additional layer of safety in daily life [12].

In this context, and as an extension of the role of service animals in supporting individuals with health conditions that require continuous vigilance and early warning, Diabetes Alert Dogs (DADs) have been introduced as a complementary resource to support glycemic monitoring and enhance quality of life for individuals with diabetes. These specially trained dogs can detect significant fluctuations in blood glucose levels, particularly during episodes of hypoglycemia and hyperglycemia, by recognizing physiological changes in their handlers [13]. DADs are conditioned to exhibit specific alert behaviors when glucose levels deviate from the expected range, enabling timely awareness and intervention without relying exclusively on invasive procedures or user-operated devices [14]. When properly trained to identify these changes through olfaction, DADs may function as a non-invasive adjunct to conventional monitoring strategies, potentially mitigating some of the limitations associated with technological failures, user fatigue, and challenges faced by children, older adults, and caregivers, while also contributing to improvements in perceived safety and overall quality of life among individuals with T1DM [15]. Nevertheless, existing evidence indicates that the quality and consistency of DAD detection are influenced by multiple factors beyond the training protocol itself, including proximity to the handler, prior dog–owner relationships, and the age at which training is initiated, among other variables [14].

Taken together, the limitations of conventional glycemic monitoring methods, combined with the growing interest in DADs as a complementary support strategy, highlight the need for a comprehensive synthesis of the existing evidence. Although a growing number of studies have explored the use of DADs in glycemic monitoring, the literature remains fragmented and heterogeneous with respect to study design, training protocols, outcome measures, and reporting standards, which hampers knowledge consolidation and limits the translation of findings into clinical practice and supportive care strategies for individuals with T1DM. Based on this rationale, the present study was designed as a scoping review aimed at mapping and synthesizing the available evidence on the role of DADs in glycemic monitoring among individuals with T1DM, with the objective of characterizing existing studies, summarizing reported outcomes, and identifying current knowledge gaps to inform future research and the potential integration of DADs as a complementary tool in diabetes management.

## 2. Materials and Methods

This scoping review followed the recommendations of the Joanna Briggs Institute (JBI) guidelines [16] and was reported according to the PRISMA-ScR [17]. To ensure thorough documentation, the study’s protocol was registered on the Open Science Framework (OSF) and is available at https://doi.org/10.17605/OSF.IO/TV2WD. The investigation adhered to a systematic, transparent methodology, encompassing the formulation of a comprehensive search strategy deployed across multiple databases. This included selecting relevant studies, extracting data, and synthesizing findings. A PRISMA-ScR checklist is included as Supplementary Material for reference.

### 2.1. Research Strategy

The search strategy was conducted in the PubMed, Scopus, and Web of Science databases, with no time restrictions. Each database had its own specific search strategy. The Boolean operators “AND” and “OR” were used to combine descriptors related to T1DM, DADs, hypoglycemic, and hyperglycemic (Supplementary Material—Table S1). Additionally, the principal author performed a manual search to validate the search strategy.

### 2.2. Eligibility Criteria

The eligibility criteria for this scoping review were defined based on a conceptual framework centered on the use of DADs as a complementary strategy for glycemic monitoring in individuals with T1DM. The central aim was to map and synthesize the available evidence on the role of trained service dogs in detecting glycemic fluctuations and their potential implications for patient safety and quality of life. In this sense, eligibility criteria were established using the Population–Concept–Context (PCC) approach recommended by the JBI. The Population comprised DADs, defined as trained service dogs involved in glycemic alert activities. The Concept encompassed DADs as service dogs trained to detect and alert hypoglycemic and/or hyperglycemic episodes in individuals with T1DM, including pre-symptomatic alerts. This concept also included outcomes related to alert performance, such as sensitivity, specificity, accuracy, alert lead time, and false alert rates, as well as reported benefits, limitations, and challenges associated with the use of DADs. The Context included clinical and non-clinical environments, such as home, school, community, workplace or outpatient settings, and mixed contexts, encompassing observational and experimental studies that described the use of DADs, their alert performance, and practical aspects related to this practice. Based on this framework, the guiding research question of the present scoping review was, “**What evidence is available regarding the relationship between DADs and glycemic monitoring in individuals with T1DM?**”.

#### 2.2.1. Inclusion Criteria

Studies were eligible for inclusion if they involved trained service dogs used to detect or alert to hypoglycemic and/or hyperglycemic events, including pre-symptomatic alerts and warnings occurring during glycemic episodes. Eligible studies were required to report at least one outcome related to glycemic detection, such as sensitivity, specificity, accuracy, alert lead time, number of events detected or missed, reduction in severe episodes, avoidance of interventions, or impacts on patient safety and quality of life. Studies conducted in both clinical and non-clinical settings, including home, school, workplace, outpatient, community, or mixed environments, were considered. In addition, preclinical or laboratory-based studies performed under controlled conditions were included if they did not involve human end-users.

#### 2.2.2. Exclusion Criteria

Studies were excluded if they consisted of secondary literature (including systematic or narrative reviews, dissertations, theses, or technical reports), did not involve individuals with T1DM or failed to provide data specific to this population, evaluated dogs trained to detect medical conditions other than diabetes, or involved dogs described exclusively as emotional support animals, without a defined task-trained role in glycemic alerting, or focused solely on training dogs to identify glucose in biological samples without a clearly defined clinical application involving human users.

#### 2.2.3. Study Selection

Initially, a comprehensive search was conducted across the previously specified databases (PubMed, Scopus, and Web of Science), and all retrieved records were imported into the Rayyan platform (Rayyan Systems Inc., Doha, Qatar) [18] for study management. Duplicate records were identified and removed prior to the screening process. In the first screening stage, titles and abstracts were independently and blindly assessed by two reviewers to evaluate eligibility according to the predefined inclusion and exclusion criteria. Studies deemed potentially relevant proceeded to the second stage, in which full-text articles were retrieved and assessed for final inclusion.

**Figure 1:**
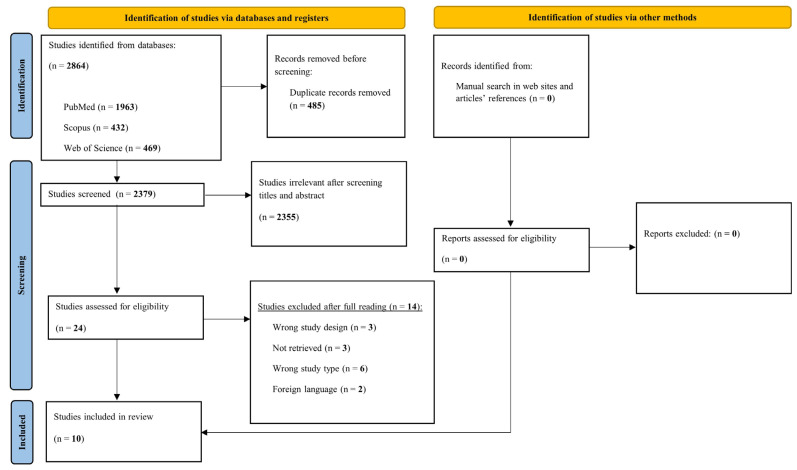
Scoping review flowchart. Source: PRISMA-ScR [17].

During the full-text evaluation, studies that did not meet the eligibility criteria were excluded, and the reasons for exclusion were systematically recorded (Supplementary Material—Table S2). Any disagreements between reviewers at any stage of the selection process were resolved through discussion and consensus. The overall process of study identification, screening, eligibility assessment, and inclusion is illustrated in the PRISMA-ScR flow diagram (Figure 1).

#### 2.2.4. Data Extraction

**Figure 2:**
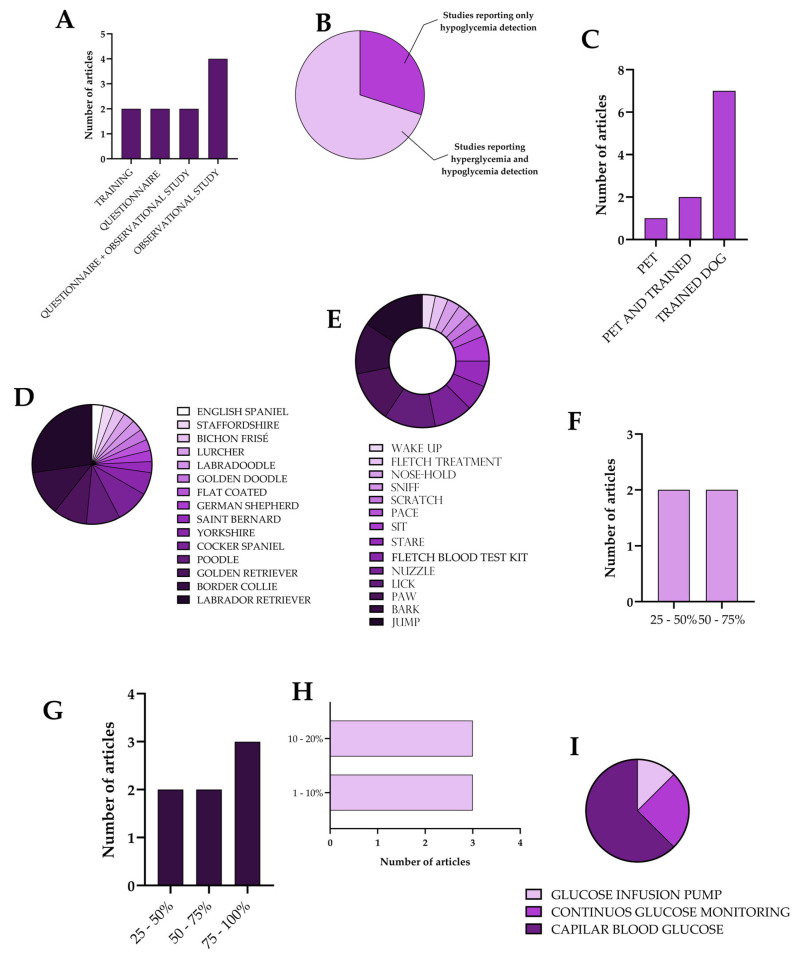
Descriptive statistical analysis of the studies included in this scoping review. All panels summarize data based on the number of studies reporting each characteristic. The following parameters are presented: (**A**) study type; (**B**) type of glycemic alteration analyzed; (**C**) relationship between dogs and their handlers; (**D**) dog breeds reported across studies; (**E**) alert behaviors reported across studies; (**F**) reported sensitivity for hyperglycemic episodes; (**G**) reported sensitivity for hypoglycemic episodes; (**H**) reported false-alarm rates; (**I**) methods used to confirm DAD alerts. For panels (**D**,**E**), a single study may contribute to more than one category, as individual studies may involve multiple breeds and/or multiple alert behaviors.

Relevant data from the studies included in this scoping review were systematically extracted and organized using a standardized data-charting form developed a priori. The extracted information was compiled and summarized in a detailed table (Table 1), which included the following variables: first author, year of publication, country of origin, study design, characteristics of the DADs (e.g., breed and prior relationship with the handler), type of glycemic alteration detected (hypoglycemia and/or hyperglycemia), alert behaviors, confirmation method of glycemic events (e.g., capillary blood glucose testing, continuous glucose monitoring, or insulin pump data), reported alert performance outcomes (such as sensitivity, alert lead time, and false alert rates), and reported impacts on safety, independence, or quality of life. To enhance clarity and facilitate comparison across studies, key descriptive characteristics and interrelationships identified in the extracted data were synthesized and visually presented using graphical representations based on descriptive statistics (Figure 2). These visual summaries aimed to highlight patterns related to study design, glycemic alteration detection, alert performance, and contextual factors influencing the use of DADs.

In addition, a narrative synthesis was conducted to contextualize and interpret the extracted data, enabling comparative analyses across studies and the exploration of methodological heterogeneity, consistencies, and discrepancies in reported outcomes. This qualitative synthesis identified knowledge gaps and emerging themes in the literature. Throughout this process, data interpretation was informed by iterative discussions among the reviewers to ensure consistency, transparency, and alignment with the objectives of this scoping review.

## 3. Results

The database searches yielded 2864 records. After duplicate removal (*n* = 485), 2379 titles and abstracts were screened, 24 full-text articles were assessed for eligibility, and 10 studies met the inclusion criteria and were included in this scoping review (Figure 1). Details of the 14 excluded studies, along with their respective justifications, are available in the Supplementary Material (Supplementary Material—Table S2).

Among the articles included in this scoping review, most were published between 2017 and 2019 (3/10 studies), while the remaining publications were from 2008 (1/10 studies), 2013 (1/10 studies), and 2015 (2/10 studies). Regarding geographical distribution, studies were clearly concentrated in the Northern Hemisphere. The United States of America accounted for the most publications (5/10 studies), followed by the United Kingdom (4/10 studies) and Canada (1/10 studies).

Figure 2 depicts the descriptive statistics for the studies included in this scoping review. A substantial degree of heterogeneity in study design was observed across the included publications, with some studies employing multiple methodological approaches within a single investigation. Apart from studies specifically focused on training service dogs to detect hypoglycemic and hyperglycemic episodes (2/10 studies), most of the included articles did not involve researcher-led experimental interventions. Instead, most studies used observational designs to document DAD alerting behaviors in real-world settings. Two studies relied exclusively on self-reported questionnaire data collected from individuals with T1DM who used service dogs for glycemic monitoring, primarily capturing perceptions of alert reliability, safety, and quality of life. An additional two studies combined questionnaire-based data with direct observational assessments of dogs’ alerting behaviors following their integration into the owners’ daily routines. The largest proportion of studies (4/10 studies) employed observational methodologies, in which DAD behavior was monitored via video recordings or owner-maintained activity logs, allowing longitudinal observation without active researcher manipulation (Figure 2A).

With regard to the glycemic alterations detected by DADs, the findings across the included studies were more homogeneous than those related to study design (Figure 2B). Among the 10 studies analyzed, the majority (7/10) reported that DADs were trained to alert to both hypoglycemic and hyperglycemic episodes. In contrast, the remaining studies (3/10 studies) described detection restricted to episodes of low blood glucose. Notably, none of the included studies reported isolated detection of hyperglycemia. This distribution may be associated with differences in training focus and reported study objectives, rather than reflecting generalized conclusions about canine detection capabilities. Hypoglycemia is commonly prioritized during DAD training due to its acute and potentially life-threatening consequences [26], which demand rapid recognition and intervention. As reported in the included studies, training programs frequently emphasize volatile organic compounds associated with hypoglycemic states, whereas hyperglycemia detection was less consistently addressed or reported.

Regarding the characteristics of the human–dog relationship described in the included studies, notable differences were observed between participants diagnosed with T1DM and their dogs (Figure 2C). In the majority of articles (7/10 studies) the dogs had undergone formal training in accordance with institutional protocols before integration into the individual with T1DM’s daily routine. Two studies included mixed groups comprising dogs obtained from training centers and pet dogs that were subsequently trained to perform DAD tasks. In these studies, dogs with prior companionship experience were reported to display different alerting patterns than dogs without such a background, which the original authors attributed to greater familiarity with the owners’ daily routines and behavioral cues. Only one study assessed dogs without formal DAD training, focusing on their behavioral responses to significant glycemic fluctuations. In this context, these dogs were described as showing lower frequencies of alert behaviors, and the findings suggested, within the scope of the original study, that familiarity and close daily coexistence with a person with T1DM may be associated with the spontaneous recognition of glucose-related physiological changes. Moreover, the data summarized in Table 1 provide a detailed overview of key characteristics of DAD use across the included studies, highlighting patterns in training background and methodological variability rather than performance validation. Most studies evaluated dogs that were not previously household pets (7/10 studies), reflecting the predominant reliance on formally trained service dogs obtained from specialized training programs. Nevertheless, three studies included dogs with prior pet status or mixed backgrounds, allowing descriptive comparisons regarding the influence of prior dog–owner relationships on reported alert behaviors.

Despite substantial heterogeneity in dog breeds across the included studies, most of which involved multiple breeds, the predominance of Labrador Retrievers, Border Collies, Golden Retrievers, and Poodles was evident (Figure 2E). These breeds are commonly described in the literature as presenting docile temperaments, high trainability, and strong human–dog bonding characteristics, traits that are frequently cited in training programs for task-oriented service dogs and reported by the included studies as relevant to their selection for use with individuals living with T1DM [27] (Figure 2D). Another outcome that demonstrated considerable heterogeneity was the specific behavioral response of DADs to glycemic fluctuations in individuals with T1DM. Such variability has been reported across studies and may reflect differences in training approaches and institutional protocols rather than standardized alert patterns. Across the included literature, the most frequently reported alert behaviors consisted of actions aimed at capturing the handler’s attention or establishing direct physical contact (e.g., barking, jumping, pawing, and licking), as described by the original authors, and were documented as the primary forms of signaling used within each study context.

The sensitivity outcomes for hyperglycemia (Figure 2F), hypoglycemia (Figure 2G), and false-alarm rates (Figure 2H) as reported across the included studies indicate that DADs were more frequently reported to be alert to hypoglycemic episodes than to hyperglycemic episodes; none of the reported sensitivity values were described as being below 25% within the context of the individual studies. Additionally, false-alarm rates were reported to remain below 20% across the included investigations, according to the definitions and confirmation methods adopted in each study, without implying standardized performance thresholds. These patterns have been described in the literature in relation to training priorities, particularly the emphasis placed on hypoglycemia detection during DAD training, given the acute and potentially life-threatening nature of low blood glucose episodes. Lastly, false-alarm rates, when reported, varied across studies and study designs, as summarized in Table 1. Lower reported false-alarm rates were observed in studies employing CGM-based or structured confirmation procedures, whereas higher reported values tended to appear in studies focused on training contexts or early-stage evaluations, highlighting the influence of methodological differences rather than generalized performance characteristics.

In studies conducted in real-world settings involving individuals with T1DM, alerts generated by DADs were reported to be compared with established blood glucose measurement methods, which served as reference measures within each study context (Figure 2I). Among the studies reporting confirmation procedures, capillary blood glucose testing was the most frequently employed method (5/8 studies), while CGM systems were used as the confirmation method in two studies (2/8 studies). In one study, confirmation relied on data obtained from the participant’s insulin pump (1/8 studies), which integrates glucose readings with insulin delivery information as described by the original authors. The use of different confirmation methods reflects the diversity of monitoring contexts and highlights the heterogeneity of methodological approaches adopted across the included studies, rather than indicating standardized procedures for evaluating DAD alert outcomes.

Overall, the findings of this scoping review indicate a pronounced geographic concentration of research on DADs in countries of the Northern Hemisphere, reflecting structural, cultural, and institutional factors that influence both the availability of trained dogs and the feasibility of systematic evaluations. Although heterogeneity in dog breeds and alert behaviors is expected and intrinsic to canine-assisted interventions, the variability observed across study designs, training protocols, outcome measures, and reporting practices represents a relevant methodological limitation of the current literature, hindering cross-study comparability and limiting the broader interpretation and transferability of findings related to the use of DADs in glycemic monitoring among individuals with T1DM.

## 4. Discussion

**Figure 3:**
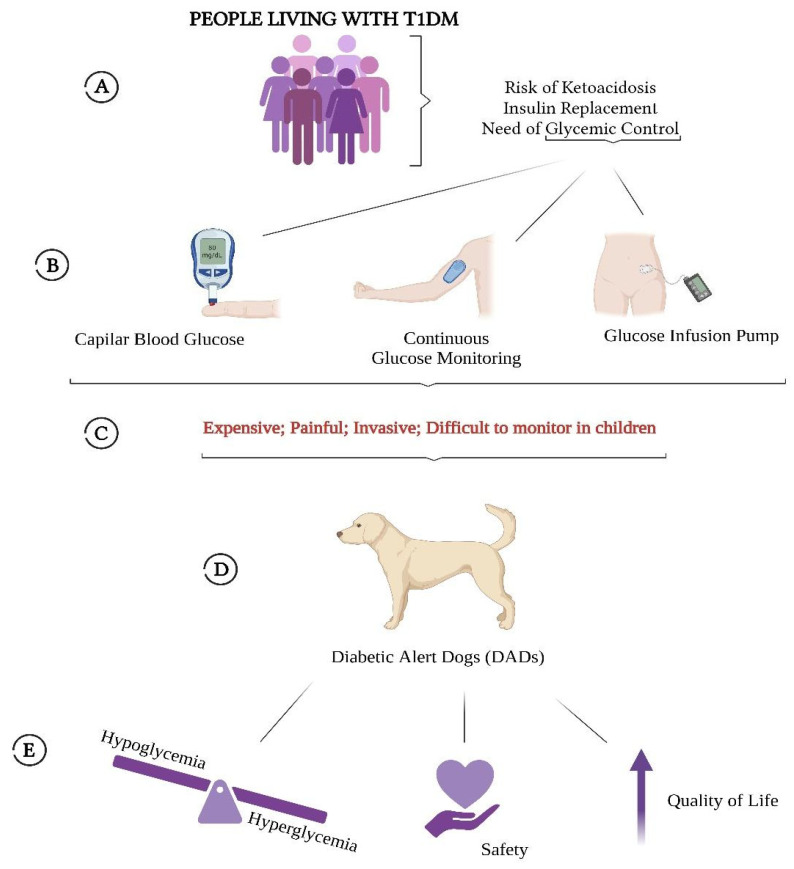
(**A**) The population targeted by this review is patients diagnosed with and living with T1DM, who require good glycemic control to administer correct insulin doses and avoid acute disease-related problems such as ketoacidosis; (**B**) Most common methods of glycemic control currently used; (**C**) Problems of those methods; (**D**) A non-invasive solution to these glycemic control methods, Diabetic Alert Dogs (DADs); (**E**) The benefits brought by DADs for patients living with T1DM.

T1DM is an autoimmune disorder characterized by immune-mediated destruction of pancreatic β-cells, ultimately resulting in an absolute deficiency of endogenous insulin production [28]. The absence of endogenous insulin production necessitates exogenous insulin administration, which contributes to pronounced glycemic variability throughout the day in individuals with T1DM. When glycemic control is inadequate, this instability can precipitate acute complications, including diabetic ketoacidosis [29]. The most common method of glycemic monitoring is capillary blood glucose testing, which, although effective for informing insulin dose adjustments, does not detect asymptomatic or nocturnal hypoglycemic episodes. Additionally, as an invasive procedure, its frequent use poses a significant challenge for parents or caregivers of children with T1DM, given the need for continuous monitoring [30]. With recent technological advances, automated continuous glucose monitoring systems have become widely available; however, despite offering substantial improvements in convenience and real-time glycemic assessment, these devices remain costly to acquire and maintain due to the need for regular sensor replacements [31]. Early observational studies reported that dogs displayed distinctive behavioral changes when their owners with T1DM experienced hypoglycemic episodes. Although the underlying detection mechanisms were not yet understood or empirically evaluated at that time [32], these initial findings laid the groundwork for the development of what are now known as DADs. Figure 3 summarizes the contextualization of this review.

### 4.1. Training Protocols and Methodological Considerations in DADs

Service dogs are specifically bred, trained, and conditioned to assist individuals with a wide range of disabilities. These include visible impairments, such as vision or mobility loss, as well as non-visible conditions, including depression, autism spectrum disorders, and neurological disorders associated with seizures or syncope [33]. The canine olfactory system is widely described in the literature as highly developed, with hundreds of millions of sensory neurons and markedly greater sensitivity than the human olfactory system, enabling fine odor discrimination [34]. Physiological changes associated with hypoglycemia in individuals with T1DM have been reported to include the production of volatile compounds, sometimes described as a fruity or sweet odor on the breath and body [35]. Based on these observations, previous studies have proposed that dogs may be able to detect volatile organic compounds emitted during glycemic fluctuations in individuals with T1DM. This hypothesis has been cited as the conceptual basis for the development of training approaches aimed at conditioning dogs to alert to glycemic changes, rather than as evidence of inherent detection efficacy.

Two studies included in this scoping review focused specifically on the training of dogs to detect glycemic fluctuations in individuals with T1DM, providing a detailed description of controlled training protocols and conditioned alert behaviors as reported by the original authors. In the experimental study conducted by Reeve et al. [19], dogs were trained using breath samples collected under defined glycemic conditions (hyperglycemia, normoglycemia, and hypoglycemia). Each participant provided six samples (two per glycemic state), which were subsequently presented to Border Collies in a structured setting. Distinct conditioned responses were established for each glycemic condition: hypoglycemia detection required sustained contact with the sample tube for more than five seconds using the nose or chin, whereas hyperglycemia and normoglycemia were signaled by a sitting response. Correct responses were consistently reinforced through food rewards, underscoring the role of positive reinforcement in shaping alert behaviors. In contrast, the study by Hardin et al. [20] employed a different training strategy, focusing on samples obtained exclusively during normoglycemic and hyperglycemic states and incorporating both breath and sweat samples, with the latter collected using a specially designed absorbent material. Although the conditioned alert behavior differed from that described by Reeve et al., the underlying reinforcement strategy remained consistent, relying on food-based rewards to strengthen the association between olfactory cues and the expected response. As discussed by the original authors, variability in conditioned alert behaviors reflects differences in training design and study protocols, rather than representing standardized or comparable measures of detection performance.

Other studies included in this review that described training procedures reported broadly similar methodological approaches, characterized by the use of biological samples collected from individuals with T1DM and the application of positive reinforcement to consolidate alert behaviors. Across the included literature, training protocols were described as varying with respect to sample type, conditioned responses, and experimental design, while consistently relying on olfactory cue discrimination and reinforcement-based learning mechanisms as reported by the original authors. This descriptive convergence highlights common elements in training approaches without implying standardized effectiveness and underscores the need for improved consistency and transparency in training descriptions and reporting practices to facilitate cross-study comparison.

Both studies conducted by Gonder-Frederick et al. [15,21] assigned DADs to individuals with T1DM through the same United States training institution, a strategy the authors described as intended to reduce variability related to differences in training programs. After placement, the institution was reported to continue providing reinforcement training and support, remaining available to address owners’ questions, as part of the ongoing training framework described in the studies. Furthermore, in addition to training to detect glycemic fluctuations, Los et al. [22] reported that dogs underwent structured socialization training, designed to habituate them to common environmental stimuli, including loud noises, the presence of children, and interactions with individuals other than their primary handlers. This component of training was described as relevant to the contexts in which DADs are expected to operate, given that they may be exposed to dynamic and unpredictable environments. By familiarizing dogs with such conditions, socialization training was reported to be intended to support the consistency of alert-related behaviors across different real-world settings, without implying standardized or validated performance outcomes.

Taken together, the evidence mapped in this section highlights a degree of descriptive convergence in the core training principles used to prepare DADs, despite variations in specific conditioned behaviors and experimental designs. Across the included literature, training protocols were consistently described as relying on olfactory cue discrimination, reinforced through positive reinforcement strategies, often reported in conjunction with ongoing post-placement support and socialization training. This pattern indicates that, within the studies included, training approaches were not centered on a single standardized alert behavior but rather on structured learning frameworks integrating scent recognition, reinforcement, and exposure to environmental stimuli, without implying uniform effectiveness across settings. At the same time, the absence of universally adopted training and reporting standards represents a relevant gap in the literature, underscoring the need for greater harmonization to facilitate cross-study comparison and replication.

### 4.2. Influence of Prior Dog–Owner Relationship on DAD’s Performance

The relevance of structured training for the development of DADs is frequently discussed in studies in which the dogs had no prior relationship with the individuals with T1DM for whom they were trained. In these investigations, training protocols were described as enabling the conditioning of alert-related behaviors, as reported by the original authors, even in the absence of a pre-existing dog–owner bond. At the same time, comparative observations across studies indicate that factors such as prior dog–owner relationships and baseline behavioral characteristics may influence how alert behaviors are expressed, highlighting a multifactorial interaction between formal training approaches and social bonding, rather than implying standardized or uniform detection performance.

A study reported that 5 of the 27 DADs evaluated had been household pets prior to receiving formal training at a specialized center in the United Kingdom [14]. According to the authors, these five dogs were described as exhibiting more consistent alert-related behaviors than the others in relation to episodes of hyperglycemia in their owners, and three of them were reported to show higher alert frequencies for both hyperglycemic and hypoglycemic events compared with dogs without prior companionship. The authors proposed that these observations might be associated with a more established dog–owner bond, suggesting that variations in alert-related behaviors may be influenced not only by olfactory discrimination training but also by individual interaction patterns and behavioral cues in people with T1DM. Complementary observations were provided by Wells et al. [13], who investigated the spontaneous alerting behaviors of companion dogs using owner-reported questionnaires. Among the 212 respondents, 138 reported that their dogs exhibited behaviors perceived as alerts during hypoglycemic episodes despite having received no formal training. These observations were interpreted by the authors as indicating that some dogs may respond to physiological or behavioral changes associated with hypoglycemia, without implying consistent or validated detection capability. Notably, although seventeen respondents reported owning more than one dog, alerting behavior was consistently observed in only one dog per household. This finding was discussed in the original study as suggesting potential influences of individual temperament, behavioral traits, and dog–owner interaction dynamics, rather than indicating inherent or standardized aptitude to function as DADs.

Collectively, these findings indicate that while structured training remains a fundamental requirement for the consistent development of DADs, prior companionship and the quality of the dog–owner relationship may act as performance-enhancing factors rather than standalone determinants. This interplay between formal training and relational context highlights the need for future studies to more systematically examine how bonding, temperament, and environmental familiarity influence DAD performance.

### 4.3. Influence of Breed and Behavioral Traits on Alert Performance

One of the main sources of heterogeneity among the studies included in this scoping review was the diversity of dog breeds employed as DADs, ranging from small samples, such as the two dogs included in the study by Reeve et al. [19], to a larger sample size, such as the 212 dogs reported in the study by Wells et al. [13]. Closely related to this variability was the wide range of alerting behaviors described across studies by dogs in response to glycemic fluctuations in individuals with T1DM. As breed-related behavioral traits are frequently discussed in the literature in relation to learning styles, temperament, and training responsiveness, breed characteristics and alert-response patterns are presented jointly in this section to reflect how these aspects were reported in the included studies, rather than to imply causal relationships or performance differences.

More than 400 dog breeds are currently recognized and cataloged worldwide, in addition to numerous mixed-breed animals resulting from crossbreeding [36]. This extensive genetic and behavioral diversity has been discussed in the literature as a factor that may contribute to challenges in standardization within canine-assisted interventions and is reflected in the heterogeneity reported across studies involving DADs. The most represented breeds in the analyzed studies were Labrador Retrievers, Border Collies, Golden Retrievers, and Poodles.

Among the breeds most frequently represented in the analyzed literature, Labrador Retrievers were the most common. This breed is commonly described in the literature as friendly, cooperative, and highly trainable, characteristics that are frequently cited in relation to their selection for different training approaches, rather than implying uniform responsiveness or performance outcomes [20]. In addition to being the most reported breed across studies, Labrador Retrievers are also the predominant choice among individuals who obtain DADs from specialized training centers, as reported in multiple investigations [14,15,21,23,24,25]. Across the included studies, the alerting behaviors most frequently conditioned in Labrador Retrievers included barking, pawing, jumping, nose-nudging, and licking, reflecting the responses selected in each training context.

Border Collies were also represented in several of the included studies. This breed is commonly described in the literature as highly trainable, with advanced cognitive abilities and an energetic behavioral profile [37]. In one study focusing exclusively on the DAD training process, only 2 Border Collies were included; nevertheless, the authors reported that these dogs acquired the conditioned alert responses defined in the study protocol, such as sitting to signal hyperglycemic episodes and nose-targeting responses to indicate hypoglycemia. These observations were discussed by the original authors in relation to learning characteristics, suggesting that cognitive and behavioral traits may be considered when selecting training strategies, without implying standardized effectiveness or performance advantages.

In studies that examined household companion dogs or dogs that had been family pets prior to DAD training, such as those conducted by Wells et al. and Wilson et al. [12,13] breeds including Golden Retrievers and Poodles were more frequently reported. Golden Retrievers are commonly described in the literature as showing high social tolerance toward humans and unfamiliar dogs, a trait that is frequently cited in relation to their selection for environments with frequent social interaction, rather than implying functional advantages in alert-related outcomes [20]. Poodles, in turn, often reported as being used as service dogs for individuals prone to seizures or syncope, as described in previous studies, which has been discussed in the literature in relation to their inclusion in training contexts involving loss-of-consciousness-related conditions, without establishing direct associations with hypoglycemia detection in individuals with T1DM [37]. Notably, the study by Wells et al. [13] was the only investigation to report instances in which dogs awakened from sleep to alert their owners to glycemic fluctuations. This observation was reported within the context of that specific study and was discussed by the authors in relation to prolonged cohabitation and dog–owner interaction, rather than as evidence of generalized or reproducible alert behavior. Overall, the findings were presented as reflecting the interaction between breed-related traits, individual temperament, and relational factors, as described across studies, without implying standardized performance or effectiveness of DADs.

Taken together, the findings mapped in this section suggest that breed-related traits and individual behavioral characteristics have been discussed in the literature as factors potentially associated with variations in the alert behaviors reported for DADs. While no single breed can be considered universally optimal for glycemic detection, breeds commonly selected for service roles are often described as sharing behavioral attributes such as high trainability, social tolerance, cognitive flexibility, and adaptability to diverse environments. Across the included studies, these traits were cited in relation to training selection and contextual suitability, rather than as indicators of consistent or reliable alert execution under real-world conditions. Importantly, the observed variability in alerting patterns across breeds highlights the heterogeneity of training approaches, study designs, and reporting practices, indicating that alert-related behaviors are shaped by a complex interaction of factors, including genetic background, training protocols, individual temperament, and dog–owner interaction dynamics, as described in the original studies. This complexity underscores the need for future research to move beyond breed-based categorizations and to adopt standardized yet flexible evaluation frameworks capable of capturing both biological and contextual determinants of alert behaviors without presuming performance equivalence or effectiveness.

### 4.4. Sensitivity and False-Alarm Rates in Diabetes Alert Dogs

Sensitivity in detecting glycemic fluctuations during hyperglycemic and hypoglycemic episodes has been commonly reported as a key outcome measure in the included studies when describing DAD-related alert behaviors. According to the literature included, sensitivity values were reported to vary substantially, reflecting differences in study design, training protocols, confirmation methods, and contextual factors. The study by Hardin et al. [30] reported higher sensitivity for hypoglycemia (>87%) in a study focused on the training phase of DADs. These values were reported under controlled experimental conditions, and the authors discussed that the structured environment and limited external behavioral or environmental influences may have influenced the reported results, which may not be directly comparable to observations obtained in real-world settings.

In studies conducted under applied or real-life conditions, sensitivity for detecting hypoglycemia was reported to vary across investigations and was generally higher than that for hyperglycemia within the context of each study’s design and confirmation methods. Evaluations of both hypoglycemic and hyperglycemic episodes were conducted in studies by Rooney et al. [14] and Gonder-Frederick et al. [15]. In the study by Rooney et al., sensitivity for hypoglycemia was reported to exceed 83%, while sensitivity for hyperglycemia exceeded 67%. In the same investigation, sensitivity values were also reported to remain above 50% in both conditions, at 59.2% for hypoglycemia and 56.1% for hyperglycemia, according to the authors’ criteria. The Los et al. study focused exclusively on hypoglycemia in individuals with T1DM and reported sensitivity values greater than 36% in that context. Notably, Gonder-Frederick et al. reported sensitivity values stratified by participant state (awake or asleep). Sensitivity was reported to be higher during waking hours, with rates of 35.9% for hypoglycemia and 26.2% for hyperglycemia; during sleep, sensitivity declined, reaching 22.2% for hypoglycemia and falling below 10% for hyperglycemia, as documented by the authors. Wilson et al. [25] presented a comparative analysis of CGM sensor readings and DAD alert reports, in which hypoglycemia-related alerts issued by dogs were reported alongside CGM data, with sensitivity values reported as exceeding 55%, without implying direct equivalence or superiority between the methods.

Across the included studies, higher reported sensitivity values for hypoglycemia than for hyperglycemia were consistently described, and some investigations did not report sensitivity estimates for elevated glucose levels. This pattern has been discussed in the literature in relation to training priorities, particularly the emphasis placed on hypoglycemia during DAD training due to its acute and potentially life-threatening consequences. As described by the original authors, training protocols were frequently reported to focus on the detection of volatile organic compounds associated with hypoglycemic states in breath and sweat, whereas hyperglycemia detection was less consistently addressed or reported. Although hyperglycemia is widely recognized as clinically relevant because of its association with long-term complications, the included studies primarily described DAD alerting behaviors in relation to hypoglycemic events, reflecting the focus adopted within each study context, without implying a standardized or exclusive functional role for DADs.

False-alarm rates, defined as alerts not corroborated by standard glycemic measurement methods, were reported in several studies and were described as an important complementary outcome to sensitivity measures. Among the applied studies involving DADs living with individuals with T1DM, the lowest false-alarm rate was reported in the study by Los et al. [22], with approximately 2% false alarms per day. Conversely, Gonder-Frederick et al. [15] reported higher false-alarm rates within real-world settings, reaching 7.8%. The highest overall false-alarm rate among the studies included in this review was observed in the training-focused study by Hardin et al. [20], exceeding 19% within the context of controlled training conditions. According to the included studies, reported false-alarm rates did not exceed 20%; however, these values should be interpreted within the context of study design, confirmation methods, and training stage, rather than as indicators of consistent or reliable alert performance. The observed variability highlights the influence of methodological and contextual factors on false-alarm reporting, underscoring the need for standardized definitions and reporting frameworks to facilitate comparison across future investigations.

Collectively, the evidence mapped in this section indicates that the included studies have reported the use of DADs in the detection of glycemic fluctuations, particularly hypoglycemic episodes, with substantial variability across study designs, settings, and outcome measures. Higher reported sensitivity values were more frequently described in controlled training environments, whereas studies conducted under applied or real-world conditions reported more variable sensitivity estimates, influenced by contextual factors such as human behavior, environmental complexity, and sleep–wake states, as described by the original authors. Importantly, across the included literature, hypoglycemia was more frequently reported as being detected with higher sensitivity than hyperglycemia, a pattern discussed in relation to training priorities and the acute clinical relevance of low blood glucose levels, rather than as evidence of standardized detection performance. Taken together, these observations highlight how DADs have been investigated as a complementary support strategy for glycemic monitoring, while underscoring the need for standardized definitions, performance metrics, and reporting practices to improve cross-study comparability and interpretation, without implying validated clinical effectiveness.

### 4.5. Impact of Diabetes Alert Dogs on Quality of Life and Perceived Safety

Individuals with T1DM often experience persistent concern about hypoglycemic episodes, given the potentially severe consequences of acute glucose deprivation, including loss of consciousness and diabetic coma [38]. In some cases, this fear leads individuals to intentionally maintain chronically elevated blood glucose levels as a protective strategy against hypoglycemia, a practice that increases the risk of long-term complications associated with sustained hyperglycemia [38]. Beyond this psychological burden, current glycemic monitoring methods are invasive, require sustained vigilance, and may impose significant financial costs. Taken together, these factors have been described as negatively affecting quality of life and have been discussed in the literature as contextual elements underlying interest in complementary support strategies, including the use of DADs, without implying clinical effectiveness or substitutive roles.

The potential impact of DADs on quality of life is particularly evident in pediatric populations. In the study conducted by Petry et al. [23], 63 of the 135 participants were children with a mean age of 11 years, reflecting the involvement of caregivers in the use of DADs as a complementary support strategy for glycemic monitoring in this age group. Children are especially susceptible to severe complications, including diabetic ketoacidosis and hypoglycemic coma, and nighttime monitoring represents a well-recognized challenge. Notably, when participants were asked about hospitalizations related to diabetic ketoacidosis or hyperglycemia (excluding events at diagnosis), the authors reported a marked reduction in self-reported hospitalization events after the introduction of DADs into daily care routines, as described by caregivers, without establishing a causal relationship or controlling for potential confounding factors.

Subjective perceptions of improvements in autonomy, confidence, and emotional well-being were frequently reported in adult populations as well. In the questionnaire-based study by Rooney et al. [24], twelve of the fourteen participants reported feeling more independent after acquiring a DAD, and eleven expressed high levels of confidence in the dog’s ability to alert to glycemic fluctuations. Importantly, all participants reported no regret regarding the acquisition of a DAD, and thirteen indicated a marked improvement in overall quality of life. Taken together, these self-reported outcomes indicate that, from the perspective of adult handlers, the presence of a DAD was associated with perceived psychological reassurance, emotional support, and an increased sense of safety in daily life, without implying objective validation of alert accuracy or clinical effectiveness.

Then, the evidence mapped in this section suggests that the included studies have reported perceived quality-of-life–related benefits associated with the presence of DADs for individuals with T1DM and their caregivers. According to the reviewed literature, participants reported reductions in fear of hypoglycemia, enhanced perceived safety, and greater self-reported independence, addressing psychosocial dimensions of diabetes management that are not fully captured by conventional glycemic monitoring technologies. Although these observations were primarily derived from self-reported outcomes and observational study designs, their recurrent description across different populations highlights the relevance of psychosocial factors when examining how DADs are experienced by users, rather than establishing objective benefit or effectiveness. Taken together, these findings emphasize the importance of incorporating patient-centered outcomes into future research frameworks, positioning quality of life as a relevant contextual endpoint alongside technical and methodological measures, without presuming clinical validation.

### 4.6. Limitations and Future Perspectives

While the findings synthesized in this scoping review offer a descriptive overview of how DADs have been investigated as a complementary strategy for glycemic monitoring in individuals with T1DM, it is essential to interpret these results with consideration of the limitations inherent to the existing body of evidence. As a scoping review, the present work aimed to map research trends, methodological approaches, and reported outcomes, rather than to evaluate effectiveness or establish causal relationships. Accordingly, this section provides a critical appraisal of the principal gaps and methodological constraints identified across the included studies, followed by an expert perspective focused on methodological considerations and future research directions necessary to strengthen the evidence base and inform the contextual discussion of DADs within diabetes management, without implying validated clinical integration.

Conversely, the absence of studies originating from low- and middle-income countries and from regions in the Southern Hemisphere was observed across the included literature, highlighting potential gaps in access, feasibility, and research capacity in the investigation of DADs. This geographic imbalance may limit the generalizability of the available evidence and underscores the need for future investigations conducted in more diverse sociocultural and healthcare contexts, particularly in settings where conventional glycemic monitoring technologies are reported to be less accessible, less affordable, or associated with additional practical barriers.

Beyond geographic concentration, several methodological limitations were recurrently observed across the included studies. A major limitation relates to the heterogeneity of study designs, training protocols, outcome definitions, and performance-related measures. Sensitivity, false-alarm rates, and alert lead time were not consistently reported across investigations, and confirmation methods varied substantially between studies, ranging from capillary blood glucose testing to continuous glucose monitoring and insulin pump-derived data. This lack of methodological and reporting standardization limits cross-study comparability and precludes more robust quantitative synthesis, underscoring the need to develop harmonized reporting frameworks specifically tailored to canine-assisted glycemic monitoring.

Another important gap concerns the predominance of observational designs and self-reported outcomes. While questionnaires and owner-reported data provide valuable insights into perceived safety, independence, and quality of life, they are inherently subject to recall bias and expectancy effects. Prospective controlled studies, including standardized real-world validation protocols that combine objective glycemic measurements with systematic documentation of alert-related behaviors, were infrequently reported in the literature. Additionally, most investigations involved relatively small sample sizes, further limiting statistical power and external validity.

From an expert perspective, future research on DADs may benefit from the adoption of a multipronged methodological approach. First, the development of consensus-based guidelines for training, alert behavior classification, performance evaluation, and outcome reporting has been proposed as a strategy to strengthen the coherence and interpretability of the evidence base. Second, the literature has highlighted greater consideration of contextual variables, such as sleep–wake states, daily routines, environmental distractions, and dog–owner interaction patterns, as relevant given their potential influence on the expression of alert-related behaviors. Third, longitudinal study designs may contribute to understanding the durability of training effects, changes in reported alerting behaviors over time, and the long-term psychosocial impact of DADs on both individuals with T1DM and their caregivers.

Importantly, the included literature consistently emphasizes that DADs have been discussed as complementary rather than substitutive tools in relation to conventional glycemic monitoring technologies. In this context, DADs have been described as addressing psychosocial dimensions, such as perceived safety, emotional well-being, and autonomy, which may be insufficiently captured by technological solutions alone, based on user-reported experiences. From a translational and research-oriented perspective, the potential inclusion of DADs within multidisciplinary diabetes care frameworks, alongside medical, technological, and behavioral interventions, has been proposed in the literature as an area warranting further investigation, rather than as an established or validated care model.

## 5. Conclusions

This scoping review mapped and synthesized the available literature addressing the relationship between DADs and glycemic monitoring in individuals with T1DM. Across the included studies, DADs were investigated for their ability to detect glycemic fluctuations, particularly hypoglycemic episodes, across a wide range of training approaches, breeds, and real-world contexts. Reported sensitivity estimates for hypoglycemia were generally higher than those for hyperglycemia, a pattern discussed in the literature in relation to training priorities and the acute clinical relevance of low blood glucose levels, rather than as evidence of standardized detection performance. Similarly, reported false-alarm rates varied across studies and should be interpreted considering methodological heterogeneity, confirmation procedures, and study design, without implying validated alert reliability.

Beyond technical aspects, the mapped evidence indicates that several studies have reported patient- and caregiver-perceived psychosocial outcomes associated with DADs, including enhanced perceived safety, increased self-reported independence, and improvements in quality of life. These observations were primarily derived from self-reported and observational data and were discussed as particularly relevant in pediatric populations and in contexts characterized by fear of nocturnal hypoglycemia, where conventional monitoring technologies may not fully capture the psychosocial dimensions of diabetes management.

At the same time, the existing body of literature is characterized by substantial heterogeneity in study design, training protocols, outcome definitions, confirmation methods, and reporting practices, as well as by marked geographic concentration in high-income countries of the Northern Hemisphere. These limitations restrict the generalizability of the available evidence and preclude definitive conclusions regarding effectiveness or clinical impact. Accordingly, DADs should be interpreted in the literature as complementary, context-dependent support strategies rather than as substitutes for conventional glycemic monitoring technologies or as validated clinical interventions.

Overall, this scoping review highlights how DADs have been explored in the scientific literature as a supportive component of glycemic monitoring in T1DM, while underscoring critical gaps in methodological standardization, geographic representation, and study design. Future research would benefit from harmonized reporting frameworks, broader inclusion of diverse sociocultural contexts, and well-designed prospective investigations to clarify how DADs are experienced, evaluated, and contextualized within diabetes management, without presuming clinical effectiveness or long-term impact.

## Figures and Tables

**Table 1:** Main characteristics of glucose fluctuation detection by DADs.

Author and Year	Pet or Not	Glycemic Detection	Confirmation Method	False Alarm
Wells et al., 2008 [13]	Pet	Hypoglycemia	Capillary Blood Glucose	Not Reported
Rooney et al., 2019 [14]	Both	Hyper/Hypo	Not Reported	Not Reported
Gonder-Frederick et al., 2017 [15]	No	Hyper/Hypo	Continuous glucose monitoring	7.80%
Reeve et al., 2020 [19]	No	Hypoglycemia	Not Reported	Not Reported
Hardin et al., 2015 [20]	No	Hypoglycemia	Not Reported	19.60%
Gonder-Frederick et al., 2017 [21]	No	Hyper/Hypo	Capillary Blood Glucose	Not Reported
Los et al., 2017 [22]	No	Hyper/Hypo	Capillary Blood Glucose	Not Reported
Petry et al., 2015 [23]	Both	Hyper/Hypo	Capillary Blood Glucose	2.08%
Rooney et al., 2013 [24]	No	Hyper/Hypo	Capillary Blood Glucose	14–6%
Wilson et al., 2019 [25]	No	Hyper/Hypo	Continuous glucose monitoring	4.56%

## Data Availability

The original contributions presented in this study are included in the article/Supplementary Material. Further inquiries can be directed to the corresponding author.

## Supplementary Materials

The following supporting information can be downloaded at https://www.mdpi.com/article/10.3390/medsci14010039/s1, Table S1: Search strategies for Pubmed, Scopus, and Web of Science articles; Table S2: Excluded articles and reasons.

## Abbreviations

The following abbreviations are used in this manuscript:

CNPq	Conselho Nacional de Desenvolvimento Científico e Tecnológico
DADs	Diabetic Alert Dogs
JBI	Joanna Briggs Institute
IDF	International Diabetes Federation
DM	Diabetes Mellitus
CGM	Continuous Glucose Monitoring
OSF	Open Science Framework
PRISMA-ScR	Preferred Reporting Items for Systematic reviews and Meta-Analyses extension for Scoping Reviews
T1DM	Type 1 Diabetes Mellitus
