# Supplementary material for: What Is the Role of Diabetic Alert Dogs in Glycemic Monitoring for Individuals with Type 1 Diabetes? A Scoping Review
Source: Med Sci (Basel). 2026 Jan 13;14(1):39. doi: 10.3390/medsci14010039 (PMC12821445; doi:10.3390/medsci14010039)
Supplement: Supplementary file 1 [file medsci-14-00039-s001.zip › Souza et al., 2025 - Supplementary Files.pdf]

Tables

Table S1. Search strategies for Pubmed, Scopus, and Web of Science articles.

Database	Queries
**Pubmed**	"glucose metabolism disorders"[MeSH Terms] OR "glucose metabolism disorders"[Title/Abstract] OR "Hyperglycemia"[Title/Abstract] OR "Hyperglycemia"[MeSH Terms] OR "Hypoglycemia"[MeSH Terms] OR "Hypoglycemia"[Title/Abstract] OR "blood glucose"[Title/Abstract] OR "blood glucose"[MeSH Terms] OR "low blood sugar"[Title/Abstract]<br><br>"diabetes mellitus"[MeSH Terms] OR "diabetes mellitus"[Title/Abstract] OR "T1DM"[Title/Abstract] OR "type 1 diabetes mellitus"[Title/Abstract] OR "TYPE 1 DIABETES"[Title/Abstract] OR "humans"[MeSH Terms]<br><br>"working dogs"[MeSH Terms] OR "working dogs"[Title/Abstract] OR "service animals"[Title/Abstract] OR "service animals"[MeSH Terms] OR "Canine"[Title/Abstract] OR "DADs"[Title/Abstract] OR "diabetes alert dogs"[Title/Abstract] OR "dogs"[Title/Abstract] OR "diabetes alert dog"[Title/Abstract]) NOT "diabetic dog"[Title/Abstract]
**Scopus**	(TITLE-ABS-KEY (Hyperglycemia) OR TITLE-ABS-KEY ("glucose metabolism disorders") OR TITLE-ABS-KEY (Hypoglycemia) OR TITLE-ABS-KEY ("low blood sugar") OR TITLE-ABS-KEY ("blood glucose"))<br><br>(TITLE-ABS-KEY ("working dogs") OR TITLE-ABS-KEY ("service animals") OR TITLE-ABS-KEY (Canine) OR TITLE-ABS-KEY ("DADs") OR TITLE-ABS-KEY ("diabetes alert dog") OR TITLE-ABS-KEY ("diabetes alert dogS"))<br><br>(TITLE-ABS-KEY ("diabetes mellitus") OR TITLE-ABS-KEY (T1DM) OR TITLE-ABS-KEY ("TYPE1 DIABETES MELLITUS") OR TITLE-ABS-KEY ("TYPE 1 DIABETES"))
**Web of Science**	"glucose metabolism disorders" (Topic) or "Hyperglycemia" (Topic) or "Hypoglycemia" (Topic) or blood glucose (Topic) and low blood sugar (Topic)<br><br>diabetes mellitus (Topic) or T1DM (Topic) or TYPE 1 DIABETES (Topic) or TYPE 1 DIABETES MELLITUS (Topic)<br><br>working dogs (Topic) or service animals (Topic) or Canine (Topic) or "DADs" (All Fields) or "diabetes alert dogs" (Topic) or "diabetes alert dog" (Topic) or DOG (Topic)

Table S2. Excluded articles and reasons.

Author	Article's title	Year of publication	Reason for exclusion
Woollam et al.	"Steps toward clinical validation of exhaled volatile organic compound biomarkers for hypoglycemia in persons with type 1 diabetes"	2025	Wrong study design
Koufakis et al.	"And the Dog Was Barking: Transforming Quality of Life in Diabetes Through Innovative Hypoglycemia Detection"	2025	Wrong publication type
Eason et al.	"Still "serving" us? Mutualism in canine scent detection of human illness"	2019	Wrong publication type
Siebenand et al.	"Diabetes alert dogs: Four-legged sensor"	2010	Wrong publication type
Rosenthal et al.	"Diabetes alert dogs"	2010	Not Retrieved
Los et al.	"Reliability of Trained Dogs to Detect Hypoglycemia in Type 1 Diabetes"	2016	Not Retrieved
Lippi et al.	"Hypoglycemia Alert Dogs: A Novel, Cost-effective Approach for Diabetes Monitoring?"	2016	Wrong publication type
Hardin et al.	"Hypoglycemia Alert Dogs-Innovative Assistance for People With Type 1 Diabetes"	2012	Wrong publication type
Mialet et al.	"How dogs become accurate instruments: care, attunement, and reflexivity"	2020	Wrong study design
Hügler et al.	"Diabetic alert dogs: A good nose for hypoglycemia"	2012	Foreign Language
Gonder-Frederick et al.	"Diabetic alert dogs: a preliminary survey of current users"	2013	Wrong publication type

Spake et al.	"Could a dog save your life? No one knows for sure how they do it, but a growing number of canine companions are helping people with diabetes avoid dangerous hypoglycemia"	2008	Not Retrieved
Iacobucci et al.	"Sixty seconds on . . . medical detection dogs"	2019	Wrong publication type
Spero et al.	"Dogs for Diabetes"	2016	Wrong publication type
